# Brain-Type Glycogen Phosphorylase Is Crucial for Astrocytic Glycogen Accumulation in Chronic Social Defeat Stress-Induced Depression in Mice

**DOI:** 10.3389/fnmol.2021.819440

**Published:** 2022-01-24

**Authors:** Yuanyuan Zhu, Ze Fan, Qiuying Zhao, Jiaqi Li, Guohong Cai, Rui Wang, Yi Liang, Naining Lu, Junjun Kang, Danlei Luo, Huiren Tao, Yan Li, Jing Huang, Shengxi Wu

**Affiliations:** ^1^Department of Neurobiology, School of Basic Medicine, Fourth Military Medical University, Xi’an, China; ^2^State Key Laboratory of Military Stomatology, Department of Anesthesiology, School of Stomatology, Fourth Military Medical University, Xi’an, China; ^3^Center for Brain Science, The First Affiliated Hospital of Xi’an Jiaotong University, Xi’an, China; ^4^Department of Spine Surgery, Shenzhen University General Hospital, Shenzhen, China

**Keywords:** glycogen, astrocyte, medial prefrontal cortex, brain type glycogen phosphorylase, depression

## Abstract

Astrocytic glycogen plays an important role in brain energy metabolism. However, the contribution of glycogen metabolism to stress-induced depression remains unclear. Chronic social defeat stress was used to induce depression-like behaviors in mice, assessed with behavioral tests. Glycogen concentration in the medial prefrontal cortex (mPFC) and the expression of key enzymes of the glycogen metabolism were investigated using Western blots, immunofluorescent staining, electron microscopy, and biochemical assays. Stereotaxic surgery and viral-mediated gene transfer were applied to knockdown or overexpress brain-type glycogen phosphorylase (PYGB) in the mPFC. The glycogen content increased in the mPFC after stress. Glycogenolytic dysfunction due to inactivation of PYGB was responsible for glycogen accumulation. Behavioral tests on astrocyte-specific PYGB overexpression mice showed that augmenting astrocytic PYGB reduces susceptibility to depression when compared with stress-susceptible mice. Conversely, PYGB genetic down-regulation in the mPFC was sufficient to induce glycogen accumulation and depression-like behaviors. Furthermore, PYGB overexpression in the mPFC decreases susceptibility to depression, at least partially by rescuing glycogen phosphorylase activity to maintain glycogen metabolism homeostasis during stress. These findings indicate that (1) glycogen accumulation occurs in mice following stress and (2) glycogenolysis reprogramming leads to glycogen accumulation in astrocytes and PYGB contributes to stress-induced depression-like behaviors. Pharmacological tools acting on glycogenolysis might constitute a promising therapy for depression.

## Introduction

The global prevalence of depression is increasing, causing a tremendous economic burden (Menard et al., 2016). Despite the often complete remission with current antidepressant treatment, serious side effects are common and patients require long-term therapy (Johnston et al., 2019). Mostly, a disturbed monoamine transmission is considered to be involved in the pathogenesis of depression. However, growing evidence suggests that the hypothalamic-pituitary-adrenal axis and proinflammatory cytokines also play important roles (Hayley et al., 2021; Qin et al., 2021). The fact that current treatment strategies are not useful in some patients evidences our incomplete understanding of the disease etiology. Thus, new theories are needed to improve treatment and clinical outcomes.

Glycogen, a complex glucose polymer found in a variety of tissues, normally functions as a glucose store. Glycogen has a crucial role in astrocyte energetics—maintenance of endoplasmic reticulum calcium homeostasis, managing oxidative stress, controlling the extracellular K^+^ concentration, and providing short-term energy to neurons (Dienel and Cruz, 2015; Duran et al., 2019). Numerous studies have shown that the brain glycogen metabolism is highly dynamic, finely tuned, and involved in specific neurobehavioral functions such as emotion, learning, memory, and sleeping (Bak et al., 2018). Furthermore, glycogen metabolism dysfunction is involved in the pathogenesis of depression (Detka et al., 2014). However, the specific metabolic alterations and the effects of brain glycogen metabolism on depression are still unclear.

Basal glycogen levels depend on the glycogenesis/glycogenolysis balance. The glycogen metabolism is a coordinated, multi-stage regulation system (Laurent et al., 1998). Glycogen synthase (GYS) and glycogen branching enzyme (GBE1) are responsible for cerebral glycogenesis (Malinska et al., 2020); glycogen phosphorylase (GP) is an important enzyme that regulates glycogenolysis and has three isoforms: PYGB (brain isoform), PYGL (liver isoform), and PYGM (muscle isoform) (de Luna et al., 2015). Whether these enzymes play an important role in the progression of depression is still unclear.

In the present study, we examined the behavioral effect of chronic social defeat stress (CSDS), and measured the glycogen concentration in the medial prefrontal cortex (mPFC) and the level of important enzymes of glycogen metabolism in stress-susceptible (SS) mice. In addition, we combined behavioral and genetic approaches to investigate whether astrocytic PYGB is involved in glycogen accumulation in depression.

## Materials and Methods

### Animals

Male adult wild-type (WT) C57BL/6J mice (6–8 weeks, ∼24 g) and male CD-1^®^ (ICR) mice (7–8 months, ∼45 g) were purchased from Beijing Weitong Lihua Experimental Animal Technology Co., Ltd. (China). *Pygb*^*H*11/+^ heterozygous mice were purchased from Cyagen Biosciences Inc. (China). *Pygb*^*H*11/+^ heterozygous mice were interbred to produce homozygous *Pygb*^*H*11/*H*11^ mice littermates. All mice were raised at a 12 h light and 12 h dark cycle with 22 ± 2°C temperature and 40–60% humidity, with food and water *ad libitum*.

### Chronic Social Defeat Stress

Firstly, CD-1^®^ (ICR) mice were selected based on the quality of aggressive behavior during social interactions for 180 s per day for three consecutive days (conducted once daily), and the CD-1^®^ (ICR) mice must attack C57BL/6J mice in ≥ 2 consecutive sessions. The two mice were raised in the cage with a clear perforated Plexiglas divider in the middle (26.7 cm width × 48.3 cm depth × 15.2 cm height, pore size 0.6 cm × 45.7 cm × 15.2 cm) 24 h before the defeat tests. C57BL/6J mice were attacked by CD-1^®^ (ICR) for 5–10 min, followed by C57BL/6J mice being transferred across the perforated divider to the opposite compartment, allowing for sensory contact during the next 24 h. The control mice without stress were individually placed into the same cages with the perforated divider and rotated daily; however, they were not exposed to the CD-1^®^ (ICR) aggressor. After the 10th attack, all experimental mice were kept alone for 24 h before the behavioral tests.

### Social Interaction Test

Briefly, the unstressed control mice and the attacked mice were familiarized with the environment 2 h before the experiment. Each mouse was separately placed in a square open-field area (area size 50 cm × 50 cm × 50 cm) with a small transparent plastic bottle separated from the social contact area. Next, the attacked mice were placed in the open-field area and mice mobility was videotaped for 2.5 min by a camera (Sony, SNC-VB600B, Japan). At the end, the attacked mice were removed from the open-field area, and the area immediately cleaned. Later, an unfamiliar CD-1^®^(ICR) mouse was placed into small transparent plastic bottles. The unstressed control mice or the attacked C57BL/6J mice were reintroduced into the open-field area. Mouse mobility was again videotaped for 2.5 min. Video data analysis software (Panlab, SMART V3.0, Spain) was used for data analysis. The social interaction (SI) ratio was calculated as follows: time spent by the attacked C57BL/6J mice exploring the interaction zone when the unfamiliar CD-1^®^(ICR) mouse was present in the transparent plastic bottle divided by the time when the CD-1^®^ (ICR) mouse was absent. All mice with an SI ratio below 1.0 were classified as stress-susceptible (SS), and all mice with an SI ratio above 1.0 were classified as resilient (RES). We chose SS mice for our experiments.

### Sucrose Preference Test

The SPT reflects unstressed control mice and attacked mice anhedonia. On the first day, two 50 mL water bottles were placed in each home cage for 24 h. Mice were required to adapt to the new bottle for 24 h before the beginning of the tests. On the second day, one bottle was replaced with 1% sucrose solution. The position of the bottles was switched every 8 h in case the mice had a preferred position. On the third day, the mice were deprived for 24 h of water and food to make the mice thirsty. On the fourth day, the food and bottles were put back; one bottle was filled with water, and the other with the 1% sucrose solution. During the tests, the two bottles were switched twice, and the tested mice were allowed to have a free drink for 12 h. Sucrose solution and water consumption was recorded. Sucrose preference was calculated as follows: total amount of sucrose consumed/total amount of fluid consumed.

### Tail Suspension Test

The TST was used to analyze depressive behavior. Mice tails were individually suspended on a suspension shelf using a piece of adhesive tape. Each mouse was separated by wooden boards. The mice were recorded for 3.5 min by a camera. Immobility, defined as complete motionlessness, was recorded.

### Immunoblotting

C57BL/6J mice mPFC brain tissue samples were lysed in radioimmunoprecipitation buffer. Protein concentration was assessed by a bicinchoninic acid assay kit. The protein was isolated by SDS-PAGE and transferred into polyvinylidene difluoride membranes. Then, membranes were blocked with 5% (w/v) non-fat milk for 2 h, and incubated with the following primary antibodies: rabbit anti-PYGB (1/1,000, Proteintech, 55380-1-AP, RRID: AB_11182159), rabbit anti-PYGM (1/1,000, Abcam, ab81901, RRID: AB_2174886), rabbit anti-PYGL (1/1,000, Proteintech, 15851-1-AP, RRID: AB_2175014), rabbit anti-GYS1 (1/1,000, Abcam, ab40867, RRID: AB_732659), rabbit anti-GYS2 (1/1,000, Proteintech, 22371-1-AP, RRID: AB_2879091), and mouse anti-β-actin (1/1,000, Proteintech, 60008-1-ig, RRID: AB_2289225) overnight at 4°C. To examine rabbit anti-phospho-PYGB (Ser15) (customized antibody, Gene Create Biotech), the analysis was performed as previously described (Cai et al., 2020). Then, the membranes were incubated with the corresponding secondary antibodies: goat anti-rabbit IgG (H&L) (1/10,000, Abcam, ab205718, RRID: AB_2819160) or goat anti-mouse IgG (H&L) (1/10,000, Abcam, ab205719, RRID: AB_2755049). The antibody binding was detected using chemiluminescence (Tanon, 5200 Multi, China), and analyzed with ImageJ software (ImageJ 7.0).

### Immunofluorescence

Briefly, mice were anesthetized using pentobarbital (1% i.p., 40 mg/kg weight). Then, the mice were transcardially perfused with 20 mL of pre-cold phosphate-buffered saline (PBS). The mouse organs were fixed with 40 mL of 4% paraformaldehyde (PFA). The brain specimens were post-fixed in 4% PFA for 2 h, followed by an additional 48 h of dehydration in a 30% sucrose solution at 4°C. Thick sections of 18 μm were cut coronally at the level of the mPFC region using a freezing microtome (Leica, CM 1950, Germany) at −20°C. The brain sections were permeabilized and blocked with 0.3% Triton X-100 and 3% goat serum for 2 h. Next, they were incubated overnight at 4°C with the following primary antibodies: rabbit anti-PYGB (1/200, ATLAS, HPA031067, RRID: AB_2673722) and mouse anti-S100β (1/500, Abcam, ab52642, RRID: AB_882426), followed by incubation with the corresponding secondary fluorochrome-conjugated antibodies: Alexa 488-affiniPure donkey anti-rabbit IgG antibody (1/500, Invitrogen, A21206, RRID: AB_141633), Alexa 594-affiniPure Donkey anti-mouse IgG antibody (1/500, Invitrogen, A21203, RRID: AB_141633), and Alexa 647-affiniPure Donkey anti-mouse IgG antibody (1/500, Jackson, 715-605-150, RRID: _2340862). These sections were incubated with Hoechest (1:1,000) for 10 min. A confocal laser scanning microscope (Olympus, FV1000, Japan) and confocal software (Olympus, Fluoview Ver4.2b, Japan) were used for image acquisition. Briefly, the slides were scanned under a laser confocal microscope at excitation wavelengths of 405, 488, 543, and 633 nm; and emission wavelengths of 450, 525, 590, and 670 nm. The parameters were set as follows: object lens (20×, numerical aperture = 0.75; 40 ×, numerical aperture = 0.9); sequential (line); pixel (1,024 * 1,024). All images were captured in a dark room at a temperature of 25°C. FLUOVIEW software and ImageJ software were used for image analysis.

### Electron Microscopy Analysis

The C57BL/6J mouse brains were fixed with 2% glutaraldehyde and 2% PFA for 2 h at room temperature. A vibratome (Leica, VS1000s, Germany) was utilized for preparing serial coronal sections at 50 μm thickness. Sections containing the mPFC region were collected, then rinsed by a PB buffer, and post-fixed with 1.0% osmium tetroxide for 2 h in a precooled cacodylate buffer. mPFC sections were dehydrated through an ethanol gradient from 30 to 100%, then transferred to propylene oxide, and finally embedded in Epon 812 between plastic sheets for 12 h at 60°C. These sections were cut using an ultramicrotome (Leica, EM UC6, Germany), collected on formvar-coated grids, and stained with uranyl acetate and lead citrate. Finally, they were examined using an electron microscope (JEOL LTD, 1230, Japan) at 80 kV. Images were obtained using an AMT digital imaging system (Gatan, 832 SC1000, United States).

### Periodic Acid-Schiff Staining

Mice were anesthetized, perfused, and fixed as before, then the brains were successively postfixed for 2 h and dehydrated in 30% sucrose at 4°C for 48 h, after which the brain samples were sectioned (20 μm) using a freezing microtome (Leica, CM 1950, Germany) at −20°C. After washing with PBS, the slices were blocked with 3% H_2_O_2_ in methanol at room temperature for 10 min. Next, the sections were incubated in 0.1% Triton X-100 in 0.1% sodium citrate for permeabilization for 2 min on ice. PAS staining of mouse brain tissues was performed according to manufacturer’s instructions of the PAS Stain Kit (Abcam, ab150680, United Kingdom).

### Verification of Viral Intervention at the Cellular Level

Primary astrocytes were prepared as previously described (Zhu et al., 2020). Astrocytes were collected from newborn C57BL/6J mice. Cultured astrocytes were cultured in Dulbecco’s modified Eagle’s medium (DMEM), 10% heat-inactivated fetal bovine serum (FBS), and 1% glutamine for 1 week. Then, the astrocytes were purified to remove other cells. For the experiments, astrocytes at a density of ∼ 1 × 10^5^/cm^2^ were plated on 24-well plates and cultured for 24 h at 37°C. The adeno-associated virus (AAV) serotype 9-pygb-EGFP targeting or empty vector (shNC) used for transfection with MOI was 10. After 24 h, the medium was changed to DMEM full medium. Biochemical analysis was performed 96 h later.

### Stereotaxic Surgery

To knockdown PYGB *in vivo*, we transduced mice with AAV serotype 9 with an astrocyte-specific glial fibrillary acidic protein (GFAP) promoter encoding a green fluorescent protein (EGFP) reporter together with either short hairpin RNAs targeting PYGB or shNC. To overexpress PYGB *in vivo*, we transduced mice with AAV serotype 9 with an astrocyte-specific GFAP promoter and an EGFP reporter together with AAV-pygb-EGFP targeting or shNC. AAV was purchased from GeneChem Ltd. Mice were anesthetized, and then exposed to the skull surface. The AAV (0.2 μL, 1 × 10^13^ v.g./mL) was infused into the mPFC at a rate of 0.04 μL/min. The coordinates of the stereotaxic apparatus were as follows: 1.90 mm before bregma, 0.30 mm to the left and right of the midline, and 2.26 mm deep. Behavioral testing was employed 3–4 weeks after virus delivery. Viral injection sites were verified by confirming the GFP signal in the mPFC.

### Biochemical Quantitative Assay

Mice were decapitated and 10 mg of cortex tissues of C57BL/6J mice were prepared for the test. Glycogen content was detected using a Glycogen Assay Kit (BioVision, K648, United States). GP activity was analyzed with the Glycogen Phosphorylase Activity Assay Kit (Genmed Scientifics, GMS50092.1, China); pyruvate levels were determined using the Pyruvate Colorimetric/Fluorometric Assay Kit (BioVision, K609, United States); lactate levels were determined using the Lactate Colorimetric Assay Kit II (BioVision, K627, United States); NADPH levels were determined using the PicoProbe™ NADPH Quantitation Fluorometric Assay Kit (BioVision, K349, United States); adenosine triphosphate (ATP) levels were determined using the ATP Colorimetric/Fluorometric Assay Kit (BioVision, K354, United States); and glutamate levels were determined using the Glu Assay Kit (Solarbio, BC1580, China).

### Data Analyses

Results are presented as the mean ± standard error of the mean. Statistical significance was evaluated using the Student’s *t*-test or a one-way ANOVA analysis of variance followed by the Tukey-Kramer *post hoc* test. ns: not significant; *p < 0.05, ^**^p < 0.01, ^***^p < 0.001, ^****^p < 0.0001. Mice were randomly assigned to treatment groups. GraphPad Prism 7.0 software was used for all statistical analyses.

## Results

### The Chronic Social Defeat Stress Model Induces Depression-Like Behaviors

**FIGURE 1:**
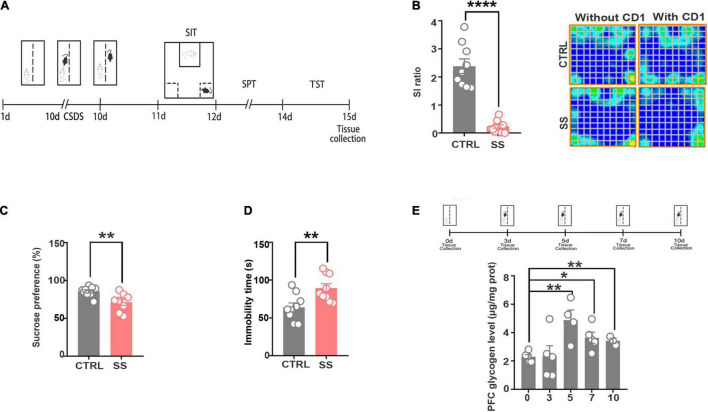
The CSDS model induces depression-like behaviors and increases cerebral glycogen levels. **(A)** Schema of the experimental design. Experimental timeline of the 10-day CSDS protocol, behavioral tests, and medial prefrontal cortex tissue collection. **(B)** CSDS induced depression-like behaviors as assessed by social interaction (*n* = 9), **(C)** sucrose preference (*n* = 9), and **(D)** tail suspension (*n* = 9) tests. **(E)** Glycogen expression significantly increased after various attack times (*n* = 5). *p < 0.05, **p < 0.01, ****p < 0.0001. CTRL, control mouse models; SS, stress-susceptible mouse models.

To explore the intermediary components modulated by neurometabolism and stress, we exposed male C57BL/6J mice to 10 days of CSDS to mimic depression. CSDS induced a depression-like phenotype (social avoidance, anhedonia) in a subset of mice, termed SS mice. Defeated mice that did not display social avoidance, as assessed using the SIT were considered RES. The SS mice showed depressive symptoms, which were validated by the SIT, TST, and SPT (Figure 1A). As shown in Figure 1B, in the SIT, the SI ratio was significantly decreased and the trajectory sparsed near the aggressor in stressed mice when compared with controls. In the SPT, sucrose preference was decreased in stressed mice (Figure 1C). Furthermore, in the TST, stressed mice had a significantly longer immobility time compared with controls, indicating aggravated depressive-like behavior (Figure 1D). These behavioral tests indicate that SS mice developed a depression phenotype after CSDS.

### Glycogen Accumulates in the Medial Prefrontal Cortex of Chronic Social Defeat Stress Mice

**FIGURE 2:**
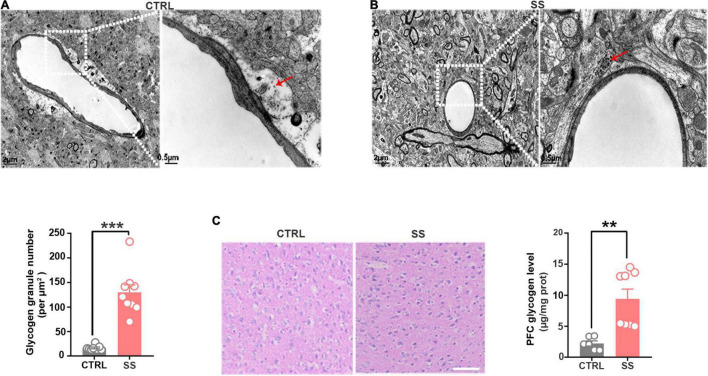
Glycogen accumulation in the mPFC. **(A)** Representative electron microscopy images of glycogen distribution in the mPFC of SS mice. Red arrows indicate glycogen granules. Scale bar = 2 μm. The scale bar in the partially enlarged figure is 0.5 μm. **(B)** Quantitative analysis of glycogen granules in SS and control mice (*n* = 9). **(C)** PAS staining images showing the glycogen distribution in the mPFC. The glycogen level was higher in SS mice compared with control mice (*n* = 6). Scale bar = 50 μm. **p < 0.01, ***p < 0.001. CTRL, control mouse models; SS, stress-susceptible mouse models; PAS, Periodic acid-Schiff.

Next, we examined the changes in glycogen content following CSDS, finding a significantly increased glycogen level in the mPFC after 5 days of CSDS (Figure 1E). On the 7th and 10th days of CSDS, the glycogen content decreased compared with that on the 5th day, though it was still much higher than in control mice (Figure 1E). Next, we detected the location of glycogen particles in the mPFC using an electron microscope. We observed significant glycogen accumulation in SS mice (Figures 2A,B). The glycogen level was significantly increased in SS mice compared with controls, as indicated by PAS staining and biochemical assays (Figure 2C).

### Astrocytic Glycogen Phosphorylase Dysfunction Is Responsible for the Extensive Glycogen Accumulation

**FIGURE 3:**
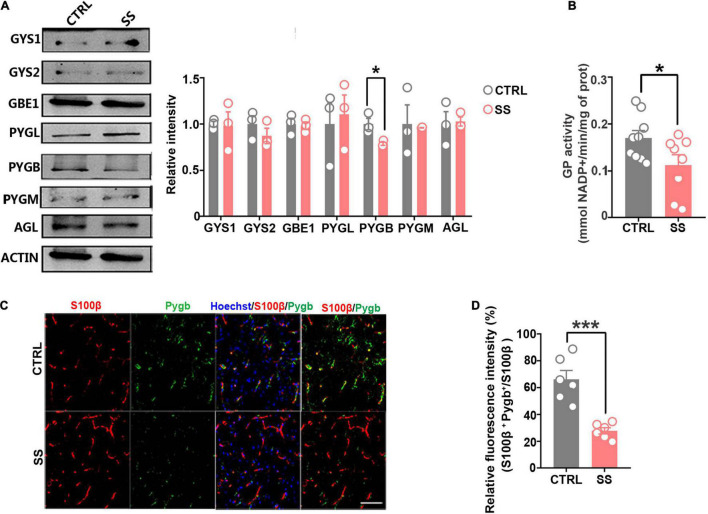
Expression of key enzymes in glycogenesis and glycogenolysis under stress conditions. **(A)** Protein levels of glycogen synthase (GYS1 and GYS2), glycogen branching enzyme (GBE1), glycogen phosphorylase (PYGB, PYGM, and PYGL), and glycogen debranching enzyme (AGL) in mice under stress. The relative optical density was calculated by dividing the density of the target band by that of the corresponding β-ACTIN band (*n* = 3). **(B)** Quantified GP activities in SS mice (*n* = 8). **(C)** Immunofluorescent double staining of S100β and PYGB, scale bar = 50 μm. **(D)** Quantification of PYGB-relative fluorescence intensity in the medial prefrontal cortex of SS mice. PYGB-relative fluorescence intensity calculated as percentage of fluorescence intensity in the colocalization area (denoted as S100β and PYGB) divided by that in the S100β^+^ area (*n* = 6). *p < 0.05, ***p < 0.001. CTRL, control mouse models; SS, stress-susceptible mouse models.

The brain glycogen metabolism depends on glycogenesis and glycogenolysis (Laurent et al., 1998). In glycogenesis, we found that the expression levels of GYS1, GYS2, and GBE1 remained stable during stress (Figure 3A). In glycogenolysis, AGL, PYGM, and PYGL levels showed no significant changes. Western blot showed that PYGB expression was decreased in SS mice compared with controls (Figure 3A), as did the total GP activity (Figure 3B). In addition, we investigated PYGB immunofluorescence in astrocytes in the SS model. We observed reduced PYGB expression in astrocytes in the mPFC of mice who underwent CSDS (Figures 3C,D).

### Augmenting Astrocytic PYGB Reduces Susceptibility to Depression

**FIGURE 4:**
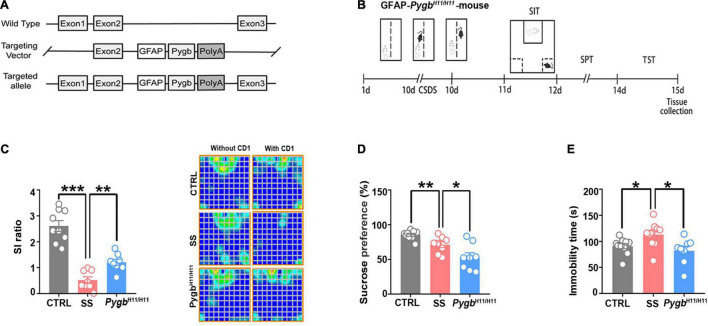
Increased astrocytic PYGB ameliorates depression-like behavior. **(A)** PYGB upregulation in *Pygb*^+/+^ mice; schematic overview of the CRISPR-/Cas-mediated genome strategy for creating GFAP-specific Pygb knock-in mice. **(B)** Schema of the experimental design of Pygb knock-in mice. Experimental timeline of the 10-day CSDS protocol, behavioral tests, and medial prefrontal cortex tissue collection. **(C)** CSDS induced depression-like behaviors as assessed by the social interaction (*n* = 8), **(D)** tail suspension (*n* = 8), and **(E)** sucrose preference tests (*n* = 8). *p < 0.05, **p < 0.01, ***p < 0.001. CSDS: chronic social defeat stress. GFAP, glial fibrillary acidic protein; PYGB, glycogen phosphorylase; CTRL, control mouse models; SS, stress-susceptible mouse models.

To assess whether depression can be alleviated by enhancing glycogenolysis at the gene level, we used an astrocyte-specific PYGB over-expression (KI-Pygb) mouse model (Cai et al., 2020). As previously reported (Fan et al., 2020), homozygous *Pygb*^*H*11/*H*11^ mice and WT littermate genotypes were identified by PCR (Figure 4A). PYGB and phosphorylated PYGB protein levels were increased in homozygous *Pygb*^*H*11/*H*11^ mice when compared with WT littermate controls (Fan et al., 2020). Behavioral analyses were performed in the CD1 attack period, according to the timeline shown in Figure 4B. The SI ratio was calculated to assess social barriers, and the social time was significantly increased in *Pygb*^*H*11/*H*11^ mice compared with the SS group (Figure 4C). Similarly, SPT and TST was decreased in *Pygb*^*H*11/*H*11^ mice compared with SS mice (Figures 4D,E).

### PYGB Knockdown in the Medial Prefrontal Cortex Increases Susceptibility to Depression

**FIGURE 5:**
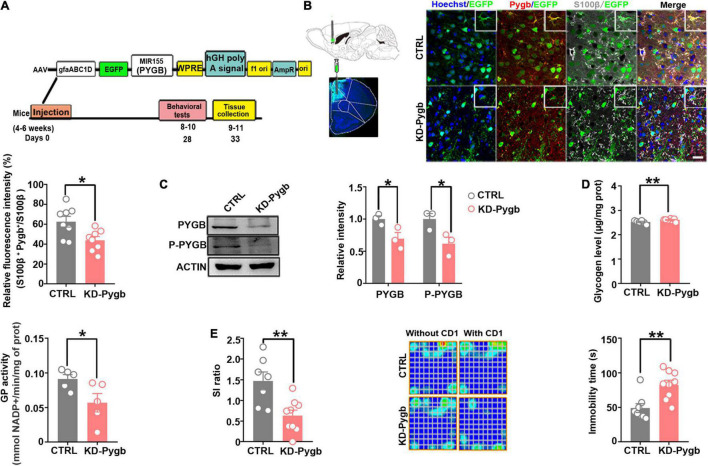
PYGB knockdown increases susceptibility to depression. **(A)** Timeline of AAV injection, chronic social defeat stress model establishment, behavioral tests, and immunohistochemistry. **(B)** Immunofluorescence staining was performed to identify the astrocyte-specific localization of exogenous PYGB in mice (*n* = 8). Scale bars = 20 μm. **(C)** Protein levels of PYGB and P-PYGB were analyzed *via* immunoblotting in the medial prefrontal cortex of Pygb-knockdown mice (*n* = 3). **(D)** Glycogen level and GP activity in astrocytic Pygb-knockdown mouse models (*n* = 5). **(E)** Pygb-knockdown mouse models were assessed by social interaction and tail suspension tests (*n* = 7). *p < 0.05, **p < 0.01. AAV, adeno associated virus; GP, glycogen phosphorylase; PYGB, glycogen phosphorylase; CTRL, control mouse models; KD-Pygb, Pygb-knockdown mouse models.

To determine whether the stress-induced PYGB decrease in the mPFC is responsible for depression-like behaviors, we knocked down PYGB expression in astrocytes in the mPFC. In addition, we injected AAV containing the astrocyte-specific GFAP promoter targeting the PYGB gene, which restricts the expression of astrocytes in the mPFC region for 4 weeks before mice performed a series of behavioral tests (Figure 5A). The knock down efficiency of PYGB verification was shown in previous studies, which demonstrated a significant increase in the glycogen level compared to control (Cai et al., 2020; Fan et al., 2020). The knockdown efficiency *in vivo* was confirmed by immunofluorescence staining and Western blot. PYGB expression was markedly decreased in astrocytes (Figure 5B), and PYGB and phosphorylated PYGB expression were clearly downregulated in Pygb knockdown mice (Figure 5C). Subsequently, we investigated the glycogen level and GP activity. The glycogen level markedly was increased in Pygb knockdown mice while GP activity was decreased (Figure 5D). Behavioral results showed that social time was markedly decreased in Pygb knockdown mice compared with controls. Similarly, the immobility time in the TST was increased (Figure 5E). However, there was no effect on sucrose preference (Supplementary Figure 1A). Considering the metabolic coupling between astrocytes and neurons, a coculture system was used that allowed the two cell types to share diffusible metabolic substrates but remain divided by a physical filter. We found that neuronal viability decreased when astrocytic PYGB was silenced by 1,4-dideoxy-1,4-imino-D-3 arabinitol (DAB, D1542, Sigma-Aldrich). The calcium activity of neurons decreased, the peak height of calcium signal increased, and the response time prolonged (Supplementary Figure 2). These results suggest that downregulation or inhibition of astrocytic PYGB in the mPFC reduces neuronal viability and increases susceptibility to depression-like behaviors.

### PYGB Overexpression in the Medial Prefrontal Cortex Decreases Susceptibility to Depression

**FIGURE 6:**
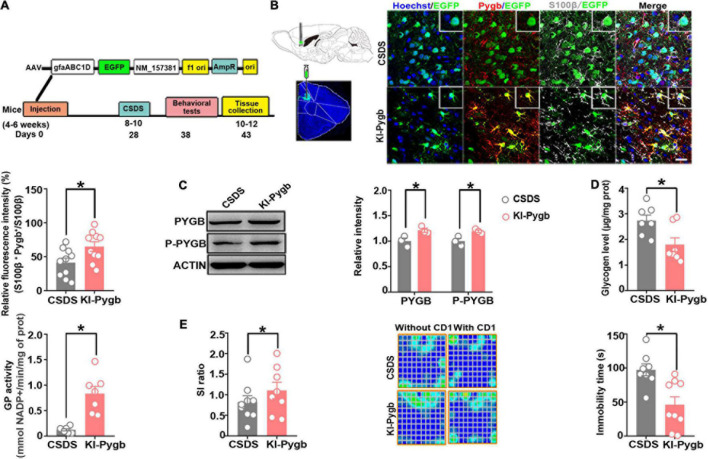
PYGB overexpression in the medial prefrontal cortex decreases susceptibility to depression. **(A)** Timeline of AAV injection, behavioral tests, and immunohistochemistry. **(B)** Immunofluorescence staining to identify the astrocyte-specific localization of exogenous PYGB in mice. Scale bar = 20 μm (*n* = 10). **(C)** Western blot and quantification of PYGB and P-PYGB protein levels (*n* = 3). **(D)** Glycogen level and GP activity in astrocytic KI-Pygb mouse models (*n* = 7). **(E)** Depression-like behaviors assessed by social interaction and tail suspension (*n* = 8). *p < 0.05. AAV, adeno associated virus; PYGB, glycogen phosphorylase; CSDS, chronic social defeat stress mouse models; KI-Pygb, Pygb-knockin mouse models.

Next, we directly increased PYGB levels in the mPFC using viral-mediated gene transfer. The knock-in efficiency *in vitro* of a knock-in (KI-Pygb) AAV containing the astrocyte-specific GFAP promoter was confirmed by immunofluorescence and Western blots in cultured astrocytes, both demonstrating a clear decrease in the glycogen level compared to that in control astrocytes (Supplementary Figure 3A). Similarly, there was a significant increase in GP activity compared to that in control astrocytes (Supplementary Figure 3B). Further, PYGB and phosphorylated PYGB levels were markedly higher than in control astrocytes (Supplementary Figure 3C). Next, we injected KI-Pygb virus into the mPFC, which increased the expression of astrocytes 4 weeks before mice were exposed to CSDS (Figure 6A), finding elevated PYGB expression in astrocytes (Figure 6B). PYGB and phosphorylated PYGB expression were clearly upregulated in mice where Pygb was overexpressed (Figure 6C). In addition, the glycogen level markedly decreased, while GP activity increased in the mice where Pygb was overexpressed compared with CSDS mice (Figure 6D). Behavioral results showed significantly increased social time in the overexpressed Pygb mice compared with the CSDS mice, and the immobility time in the TST decreased (Figure 6E). However, there were no effects on sucrose preference (Supplementary Figure 1B). These data suggest that PYGB overexpression in the mPFC decreases vulnerability to stress-induced depression-like behaviors.

### PYGB Upregulation Changes the Glutamate Level and Energy Metabolites

**FIGURE 7:**
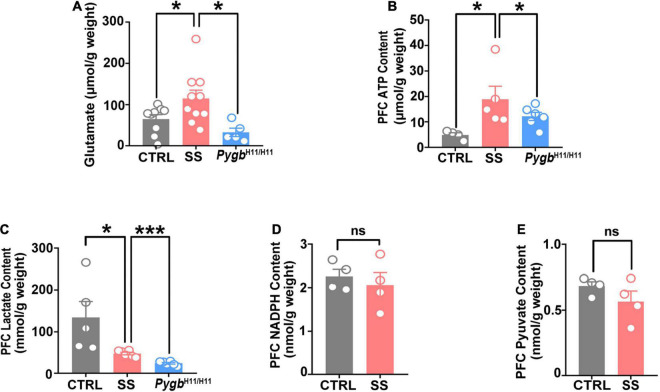
PYGB upregulation changes glutamate levels and energy metabolites. **(A)** Glutamate level in SS mice, non-stressed controls, or CSDS attacked *Pygb*^*H*11/*H*11^ mice (*n* = 5). **(B)** ATP level in SS, non-stressed control, or CSDS attacked *Pygb*^*H*11/*H*11^ mice (*n* = 4). **(C)** Lactate level in SS, non-stressed control, or CSDS attacked *Pygb*^*H*11/*H*11^ mice (*n* = 5). **(D)** NADPH level in SS and non-stressed control mice (*n* = 4). **(E)** Pyruvate level in SS and non-stressed control mice (*n* = 4). *p < 0.05, ***p < 0.001. ATP, adenosine triphosphate; CTRL, control mouse models; SS, stress-susceptible mouse models.

To further evaluate the effect of PYGB on energy metabolism and neurotransmitters, we investigated the levels of glutamate, ATP, lactic acid, NADPH, and pyruvate. An increase in glutamate and ATP occurred in SS mice, but not in non-stressed controls or CSDS attacked *Pygb*^*H*11/*H*11^ mice (Figures 7A,B). There were no differences in NADPH or pyruvate level between SS and non-stressed control mice (Figures 7D,E). Compared to non-stressed control mice, the lactic acid level was lower in SS mice and CSDS attacked *Pygb*^*H*11/*H*11^ mice (Figure 7C).

## Discussion

The present data revealed that glycogen accumulation is strongly associated with the development of depression. Our findings provide evidence that PYGB plays a substantial role in glycogen accumulation, while identifying a new target for the development of innovative antidepressants to treat major depressive disorder.

In the brain, the glycogen metabolism of astrocytes is important in physiological processes (Bak et al., 2018). Disruptions to glycogen metabolism have also been linked to learning and memory damage (Tiruchinapalli et al., 2008; Chen and Zhong, 2013). Depression is a common mental disorder with a high incidence rate, high recurrence rate, and high mortality. It is a heterogeneous syndrome consisting of several subtypes and abnormalities in multiple brain regions (Boden et al., 2021; Gold, 2021; Homberg and Jagiellowicz, 2021; Marx et al., 2021). It has been shown that adverse factors during stress lead to permanent changes in genes. These changes in genes result in raised risk coefficient of depression, diabetes, Alzheimer’s disease, hypertension, and cardiovascular disease (Graham and Smith, 2016; Sartorius, 2018; Harshfield et al., 2020; Xu et al., 2020). A recent study suggested that stress markedly increased glucose content both in the blood and brain structures (Detka et al., 2014). In addition, in the brain of stressed mouse, the main region of increased glucose is the prefrontal cortex and hippocampus (Detka et al., 2014). In our study, we used the CSDS model, a well-characterized animal model of depression (Qiao et al., 2016; Wook Koo et al., 2016; Menard et al., 2017; Jiang et al., 2018; Wang et al., 2019), and found that glycogen excessively accumulated in the mPFC.

Social defeat stress would be associated with depression-related behaviors, and the degree of cognitive deficit would differ between SS and RES mice (Huang et al., 2013; Bagalkot et al., 2015; Prabhu et al., 2019). Previous data indicate most of the damage occurred in SS and not RES mice (Menard et al., 2017), including disturbances in the metabolism of amino acids, lipids, and neurotransmitters in several brain areas. The resulting susceptibility-related metabolites provide new insights into the pathophysiology underlying stress-related mental illness (Prabhu et al., 2019). Thus, we chose the susceptible group as the experimental group rather than the resilient groups.

At the physiological level, glycogen synthesis and glycogenolysis are counterbalanced. Impairments in glycogen synthesis or glycogenolysis could change brain glycogen levels, hampering the physiological flux of glucose units through glycogen, which is important for brain function. In our results, PYGB expression, a key enzyme in glycogenolysis, was decreased in the mPFC of SS mice. The glycogen level markedly increased in PYGB knockdown mice, and PYGB knockdown in the mPFC was found to increase susceptibility to depression-like behaviors. On the contrary, augmenting astrocytic PYGB reduces susceptibility to depression. These results suggest that the main cause of increased brain glycogen levels in depression is the decrease of glycogen degradation, especially through GP.

Astrocytes play an important role in the synaptic transmission by the astrocyte-neuron lactate shuttle (Miyamoto et al., 2019). The astrocyte-derived lactate supports neuronal metabolism and synaptic plasticity. The glycogen is stored in astrocytes and rapidly converted to lactate *via* glycolysis. Glucose utilization is higher in many brain areas, and glycolysis levels are increased in animals with depression (Kanemaru and Diksic, 2009; Detka et al., 2015). Glycogen concentrations in the brain are also higher in stress models of depression (Detka et al., 2014). The results of previous studies have showed that glycogen concentrations in the frontal cortices of the prenatally stressed animals could affect the numbers of glucose transporters (Detka et al., 2014). Astrocytic glycogen accumulation-induced inadequate glycogen turnover may be the mechanism linking depression (Petit et al., 2021). Our results showed that glycogen accumulation increases depression susceptibility by depressive-like behavior test. Promoting glycogenolysis can treat corticosterone exposure-induced depression (Zhang et al., 2015).

In the brain, the energy necessary for the maintenance of proper neurotransmission results from glycogen metabolism, particularly the normal release and uptake of excitatory neurotransmitters (Chen et al., 2020). Our results showed that an increase in glutamate and ATP occurred in SS mice, but not in non-stressed control and CSDS-attacked PYGB overexpression mice. Glycogen mobilization can provide metabolic substrates to astrocytes as well as to neurons *via* the astrocyte—neuron lactate shuttle.

Brain glycogen turnover varies under different conditions; it would be interesting to investigate the pathophysiology of mood disorders by negatively impacting the glycogen turnover as well as the energy supplied to glutamatergic synapses *via* astrocyte—neuron lactate shuttle. Stress changes the milieu of neuromodulators in the brain and increases the energy expenditure of synapses (Jha et al., 2017), which can be met by the action of several neuromodulators on astrocytes that regulate glycogen turnover and lactate supply to neurons (Musazzi et al., 2019). Our results provided clues that stress disturbances can negatively impact the excitatory transmission by inadequate glycogen turnover and supply of energy substrates to synapses in susceptible individuals. The relationship between depression and transmission pathway disturbances in excitatory synapses should be explored. Glycogen is a promising therapeutic strategy for alleviating depressive symptoms by preventing impairments of energy substrates and excitatory transmission.

There were several limitations of this study. The effects of PYGB knockdown or overexpression on depression-like behavior might be due to neuronal changes; however, this requires further investigation.

## Conclusion

Taken together, our data shows that depression leads to glycogen accumulation owing to glycogenolysis reprogramming. Glycogenolysis is therefore a potential intervention target for stress; however, the specific mechanism behind its involvement in depression needs to be further studied. Specifically, PYGB was found to contribute to stress-induced depression-like behaviors, thus highlighting PYGB as a potential novel therapeutic target for the treatment of depression.

## Data Availability Statement

The original contributions presented in the study are included in the article/Supplementary Material, further inquiries can be directed to the corresponding author/s.

## Ethics Statement

The animal study was reviewed and approved by the Institutional Animal Care and Use Committee of the Fourth Military Medical University.

## Author Contributions

SW, JH, and YL conceived and designed the experiments. YZ and ZF performed the experiments, analyzed the data, and wrote the manuscript. QZ, JL, and RW assisted YZ with animal studies and histology. GC, YIL, and DL interpreted the data and performed statistical analyses. NL and JK assisted YZ with the electron microscope experiments and data analysis. SW, JH, and HT conceived this study, analyzed the data, and revised the manuscript. All authors read and approved the final manuscript.

## Conflict of Interest

The authors declare that the research was conducted in the absence of any commercial or financial relationships that could be construed as a potential conflict of interest.

## Publisher’s Note

All claims expressed in this article are solely those of the authors and do not necessarily represent those of their affiliated organizations, or those of the publisher, the editors and the reviewers. Any product that may be evaluated in this article, or claim that may be made by its manufacturer, is not guaranteed or endorsed by the publisher.
