# Supplementary material for: Brain-Type Glycogen Phosphorylase Is Crucial for Astrocytic Glycogen Accumulation in Chronic Social Defeat Stress-Induced Depression in Mice
Source: Front Mol Neurosci. 2022 Jan 24;14:819440. doi: 10.3389/fnmol.2021.819440 (PMC8820374; doi:10.3389/fnmol.2021.819440)
Supplement: Supplementary file 1 [file Image_1.pdf]

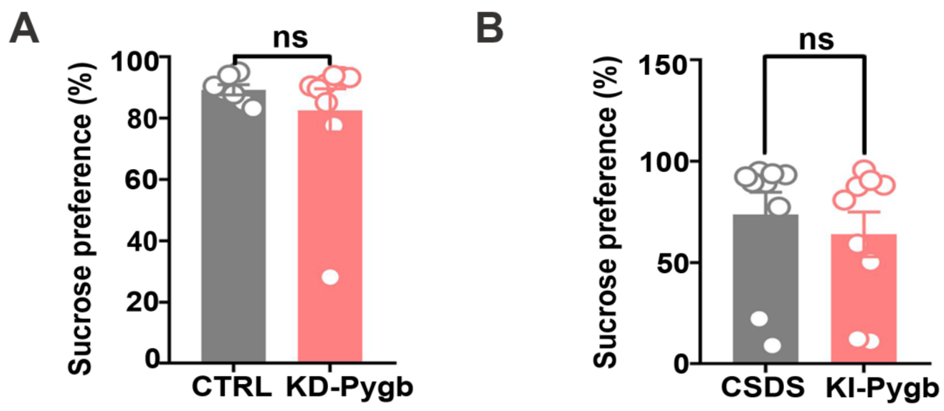

Supplementary Figure 1: Sucrose preference test in mice with PYGB knockdown or overexpression. (A) Pygb-knockdown mice models assessed by sucrose preference test (n = 7). **(B)** Pygb-overexpression mice models assessed by sucrose preference test (n = 9). ns: not significant. CTRL: control mouse models. KD-Pygb: Pygb-knock down mouse models. CSDS: chronic social defeat stress mouse models. KI-Pygb: Pygb-knock in mouse models.

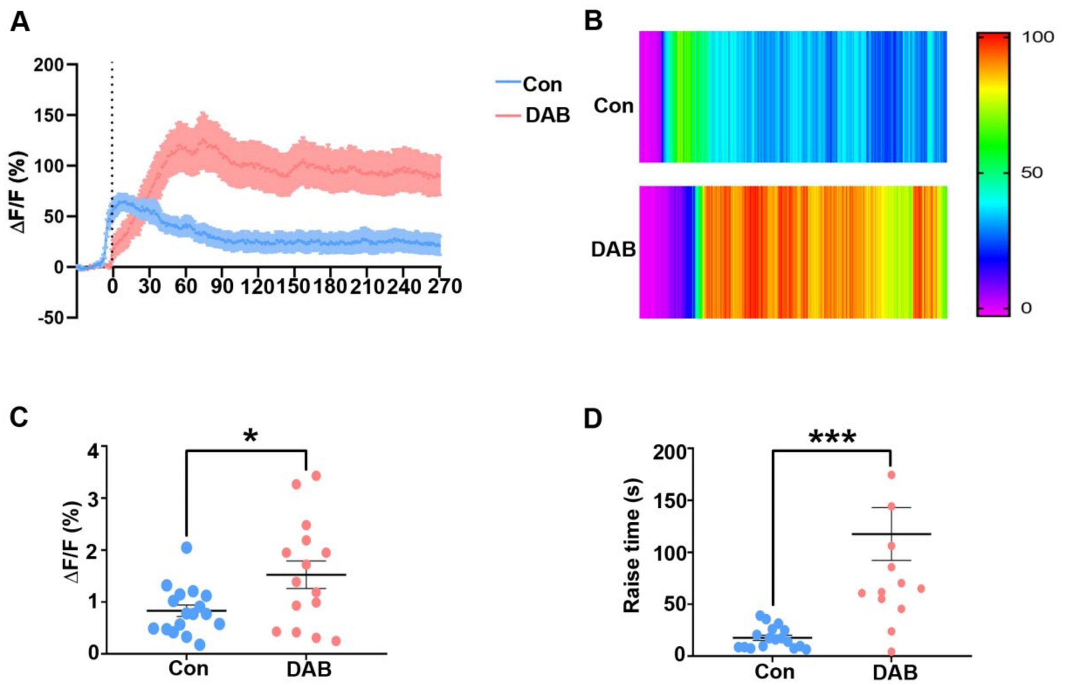

Supplementary Figure 2: Effects of knocking down Pygb expression on calcium influx. A calcium-indicator Fluo-8 AM was used to detect the intracellular Ca^{2+} oscillations in neurons. The neurons and astrocytes co-culture system was subjected to DAB (300μM) for 6 h. Neurons of Ca^{2+} imaging obtained with confocal microscopy in Con and DAB treatment group. The amplitude map (ΔF/F) (A), thermal map (B), statistical analysis of amplitude (ΔF/F) (C), and rise time (D) of the intracellular Ca^{2+} oscillations after adding ATP in each group. $^{*}p < 0.05$, $^{***}p < 0.001$.

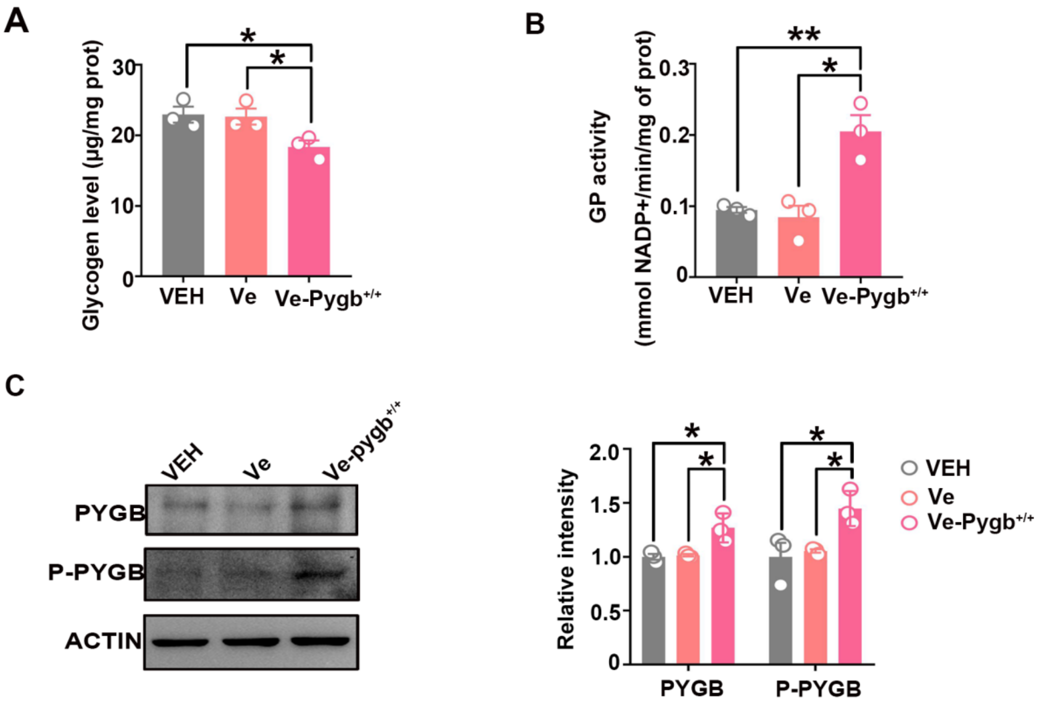

Supplementary Figure 3: Verification of successful establishment of PYGB overexpression models in cultured astrocytes. (A-B) Glycogen level and GP activity were analyzed in astrocytic PYGB overexpression models (n = 3). **(C)** PYGB protein levels were determined in astrocytic PYGB overexpression (named Ve-Pygb) models using immunoblotting (n = 3). Statistical significance was evaluated using Student's *t*-test. $^{*}p < 0.05$, $^{**}p < 0.01$. PYGB: glycogen phosphorylase.
